# Sodium MR Imaging of Articular Cartilage Pathologies

**DOI:** 10.1007/s40134-014-0041-4

**Published:** 2014-02-20

**Authors:** Štefan Zbýň, Vladimír Mlynárik, Vladimir Juras, Pavol Szomolanyi, Siegfried Trattnig

**Affiliations:** High Field MR Center, Department of Biomedical Imaging and Image-Guided Therapy, Medical University of Vienna, Waehringer Guertel 18-20, 1090 Vienna, Austria

**Keywords:** Sodium MR imaging, Cartilage, Repair tissue, Osteoarthritis

## Abstract

Many studies have proved that noninvasive sodium MR imaging can directly determine the cartilage GAG content, which plays a central role in cartilage homeostasis. New technical developments in the recent decade have helped to transfer this method from in vitro to pre-clinical in vivo studies. Sodium imaging has already been applied for the evaluation of cartilage and repair tissue in patients after various cartilage repair surgery techniques and in patients with osteoarthritis. These studies showed that this technique could be helpful not only for assessment of the cartilage status, but also predictive for osteoarthritis. However, due to the low detectable sodium MR signal in cartilage, sodium imaging is still challenging, and further hardware and software improvements are necessary for translating sodium MR imaging into clinical practice, preferably to 3T MR systems.

## Introduction

Since the intrinsic repair capacity of articular cartilage is very limited, cartilage health is very important for the health of the whole joint.

Mechanical injury is a major cause of articular cartilage destruction in young patients. Different surgical cartilage repair techniques have been developed for treatment of cartilage defects. One of the goals of these procedures is to replace the defect with a newly produced tissue that has an identical structure, composition and biomechanical properties as native articular cartilage [1]. Thus, noninvasive biochemical MR imaging methods might be useful for the evaluation of repair tissue and efficacy of different repair surgery techniques.

Osteoarthritis (OA) is a leading cause of chronic disability in the elderly population and the most common form of arthritis in synovial joints [2]. Although radiography or morphological MR imaging may be useful for assessing structural changes in knee OA and can indicate the need for knee joint replacement, both techniques are insensitive to biochemical changes in the cartilage, which occur in early stages of OA before morphological changes appear [3].

Various MR techniques have been developed for noninvasive biochemical evaluation of articular cartilage and to repair tissue. The most prominent methods are T2 mapping, delayed gadolinium-enhanced magnetic resonance imaging of cartilage (dGEMRIC) [4], T1ρ mapping [5], glycosaminoglycan chemical exchange saturation transfer (gagCEST) [6] imaging and sodium MR imaging [7]. Besides articular cartilage, sodium imaging was also used for evaluation of other musculoskeletal tissues such as the Achilles tendon [8] or skeletal muscles [9].

The goal of this review is to describe the advances in sodium MR imaging of cartilage as a potential biomarker for evaluation of OA and for examining the efficacy of cartilage repair surgery. In the following chapters, we provide a short description of the cartilage composition, an overview of sodium MR properties and sodium pulse sequences, evaluation of the glycosaminoglycan (GAG) content in cartilage, basic information and recent sodium imaging studies on repair tissue and OA cartilage.

## Composition of Normal Cartilage

Articular cartilage contains a small amount of chondrocytes (~2 % of total cartilage volume) nested in an extracellular matrix. The main components of the matrix are water, collagen and proteoglycans [10].

The relative concentration of water increases from about 65 % in the deep zone up to 80 % in the superficial zone of cartilage. About 30 % of water is trapped in the collagen fiber network; the rest appears as a gel with dissolved inorganic ions (such as sodium), and most of it can flow through the extracellular matrix by applying pressure to the cartilage [11]. High frictional resistance against water flow is one of the mechanisms that helps cartilage to withstand heavy loads.

Collagen contributes 10–20 % of cartilage wet weight. Type II collagen is the principal molecular component (90–95 % of total collagen) in hyaline cartilage and forms fibers intertwined with proteoglycans. Other collagen types are present in much smaller amounts in the matrix and help to stabilize the type II collagen network [12]. Collagen fibers are responsible for tensile and shear strength in cartilage.

Proteoglycans (PG) can exist either as protein monomers (<5 % of cartilage wet weight) or aggregates of monomers attached to hyaluronic acid fibers via specialized link proteins (5–7 % of cartilage wet weight). Each PG monomer contains one or multiple sulfated GAG side chains covalently attached to a central protein core [10]. The most common PG in cartilage is aggrecan with 100–150 GAG side chains. The GAGs contain a high concentration of negatively charged sulfate and carboxyl groups, and thus provide negative fixed charge density (FCD) to the cartilage. This results in two important physical properties of PGs. The negative FCD attracts positively charged ions (mainly sodium), and thus sodium ions are in balance with the PG content in the cartilage. Since PGs are hydrophilic, the water molecules are pumped by osmotic pressure into the cartilage. Additionally, the PG macromolecules remain separated because of the strong electrostatic repulsive force between GAGs. Through this mechanism, PGs are responsible for the compressive stiffness of cartilage [13].

## Sodium MR Properties

The sodium MR sensitivity is 9.3 % of the proton MR sensitivity, and the sodium in vivo concentration in healthy femoral cartilage is about 320 times lower than the proton one. Moreover, sodium in biological tissues exhibits very short biexponential transversal relaxation times (T2). As a result of these factors, the sodium signal-to-noise ratio (SNR) in cartilage is about 3,400 times smaller compared to the proton SNR. Thus, sodium MR imaging is challenging, and sodium images are acquired with lower SNR (10–40), lower resolution (2–5 mm) and longer measurement times (10–30 min) than proton images.

Sodium has a spin number of 3/2, which results in specific MR properties. In addition to the magnetic dipole moment, sodium also exhibits a quadrupole moment, which arises from the non-spherically symmetric distribution of the electric charge in the nucleus. When a nucleus with the quadrupole moment interacts with an electric field gradient (e.g., the gradient formed by electrons surrounding the nuclei in a molecule), a quadrupolar interaction takes place and affects the NMR properties of sodium. Based on the molecular environment of sodium, the following four motional regimes are possible: (1) fast isotropic motion, (2) slow isotropic motion, (3) slow anisotropic motion and (4) fast anisotropic motion [13]. Multiple quantum filtered sequences have been proposed to distinguish between different sodium environments. However, these sequences suffer from even lower SNR compared to conventional single-quantum sodium MR imaging [14–16]. In biological tissues, sodium nuclei can be found only in the first three regimes. Using conventional sodium MR imaging, we can only distinguish between two different sodium motional regimes—fast and slow.

Sodium in the fluid is in fast isotropic motion. The rapid tumbling of sodium ions results in a very rapid fluctuation of the electric field gradient orientation, and thus quadrupolar interaction is ‘averaged’ to zero. In this environment, both T1 (~63 ms) and T2* (~34 ms) relaxation times decay mono-exponentially.

Sodium in tissues is either in slow isotropic or slow anisotropic (ordered structures, e.g., collagen fibers) motion. In these cases, quadrupolar interaction dominates the relaxation, and T1 and T2 decay is bi-exponential, with a short T2 component (T2*_SHORT_) of ~0.9 ms, long T2 component (T2*_LONG_) of ~13 ms and two T1 components with similar relaxation times of ~20 ms. In this regime, multiple quantum filtered sequences can be used to determine the degree of order in the tissue.

## Pulse Sequences for Sodium MR Imaging

**Fig. 1:**
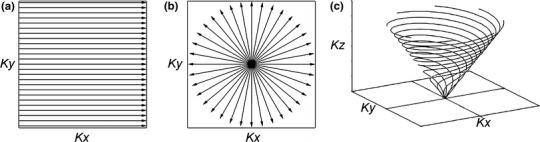
Schematic representation of **a** Cartesian, **b** radial projection and **c** twisted projection k-space trajectories

Due to the very short bi-exponential T2 values of sodium in tissues, sequences suitable for acquiring images at short echo times (TE) are useful for sodium MR imaging. The first sodium images of articular cartilage in vivo were acquired by Reddy et al. [7] using Cartesian 3D gradient echo sequences (GRE) with TE of 2 ms at 4T. The Cartesian trajectory is illustrated in Fig. 1a. In order to increase SNR in the GRE images, TE can be minimized by using a nonselective radiofrequency pulse and an asymmetric readout (partial echo). To further reduce TE in the GRE sequence, optimal gradient switching patterns together with the variable echo time (vTE) train (dynamically reduced TE toward the k-space center) [17] were recently combined in the vTE-GRE sequence [18] and used for sodium MR imaging [19]. The main advantage of Cartesian GRE sequences is their robustness.

More advanced non-Cartesian ultra-short echo time (UTE) imaging techniques can acquire sodium images with TE below 1 ms. Short TE in UTE sequences is achieved by sampling the data from the center toward the periphery of the k-space using either radial (Fig. 1b) or spiral trajectories (Fig. 1c). The most widely used type of UTE sequence for sodium MR imaging is a 3D radial projection technique [20]. The SNR of this method was further improved by the density-adapted radial projection technique, where the density of acquisition points along the projections is modified, and thus the k-space is sampled in a more efficient way [21]. Twisted projection imaging (TPI) is another approach for sodium MR imaging with efficient k-space sampling and improved SNR [22, 23]. Many other techniques, such as 3D cones [24], acquisition-weighted stack of spirals [25] and FLORET [26], were inspired by TPI. The data acquired with non-Cartesian UTE sequences are reconstructed by using regridding reconstruction [27–29] or nonuniform fast Fourier transform algorithms [30, 31]. Although the UTE sequences can provide more SNR than Cartesian sequences, they are also more prone to artifacts rising from gradient imperfections and off-resonance effects [32].

## Evaluation of GAG Content with Sodium MR Imaging

It was shown that the negative FCD of cartilage correlates with the GAG concentration of cartilage [33]. Since the negative FCD of cartilage is in equilibrium with positively charged sodium ions, sodium MR imaging was proposed to be a sensitive method for the evaluation of the GAG content in the cartilage. In early works, sodium MR spectroscopy was employed to explore the relationship between the sodium signal and PG content of enzymatically treated (trypsin, papain) cartilage [34–36]. Lesperance et al. [37] found that sodium in cartilage is 100 % MR visible, and by using the ideal Donnan theory, they estimated the FCD of cartilage from the sodium content measured by MR spectroscopy. Later studies have demonstrated that the sodium content in cartilage measured by sodium imaging is proportional to the cartilage GAG content [38–40]. Thus, sodium MR imaging can be useful for direct and noninvasive evaluation of the GAG content in native, OA cartilage and cartilage repair tissue.

**Fig. 2:**
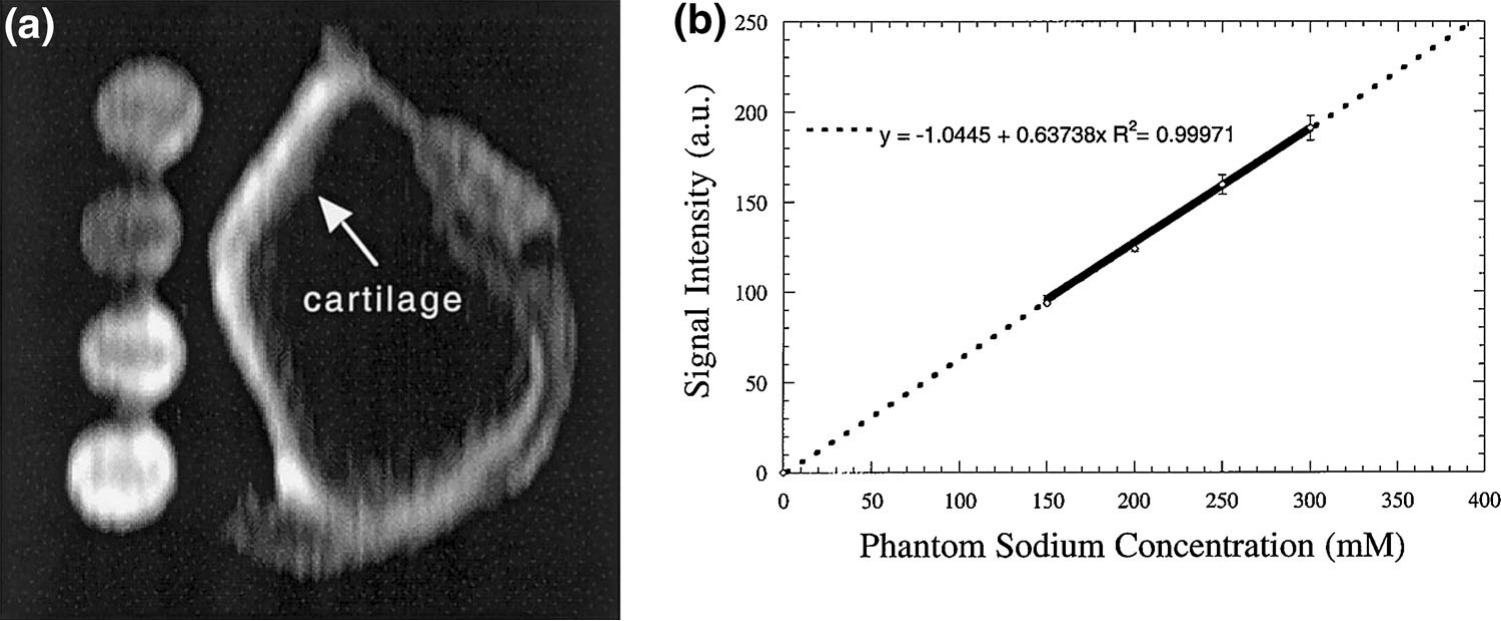
Quantification of sodium concentration in bovine cartilage. **a** Sagittal sodium 3D-GRE image of bovine patella and four saline/agarose phantoms of different concentrations (150–300 mmol/l). **b** Mean calibration curve obtained from four different slices of a 3D data set. *Circles* represent signal intensities measured from four saline/agarose phantoms. *Error bars* indicate the 95 % confidence intervals. From [41], with permission

For the evaluation of the GAG concentration and FCD in the cartilage, signal intensities from sodium images need to be converted into sodium concentration values [41]. The quantification of the sodium concentration is performed by measuring the subject together with agarose/saline phantoms of known sodium concentration (usually 100–350 mM) (Fig. 2a) and of relaxation times similar to cartilage relaxation times (usually 6–10 % agar of the phantom’s wet weight). Sodium signal intensity from phantoms corrected for their relaxation times is then plotted against their sodium concentration, and a calibration curve is obtained by linear fitting of plotted data (Fig. 2b). Sodium intensities from cartilage are then pixel-by-pixel fitted to the calibration curve to produce a tissue sodium concentration map. Since the water fraction in cartilage is about 75 %, the values in the concentration maps are divided by a factor of 0.75 [39, 40]. The sodium concentration in the healthy cartilage was found to be 240–280 mM [39, 40].

For correct evaluation of sodium concentration, sodium relaxation times in the cartilage need to be calculated. Madelin et al. [42] measured sodium relaxation times using the UTE radial sequence in knees of eight healthy volunteers at 7T. Relaxation times were evaluated in four knee cartilage regions (patellar, trochlear, femorotibial medial and femorotibial lateral cartilage), and the authors reported similar T1 (~20 ms), but different T2*_SHORT_ (0.5–1.4 ms) and T2*_LONG_ (11.4–14.8 ms) in different regions. As shown by Staroswiecki et al. [43], T2* values were not significantly different between 3T and 7T. This study also verified a linear increase of SNR with the magnetic field strength and showed about 2.3-times higher sodium SNR in cartilage at 7T compared to 3T. On the other hand, Madelin et al. [44] showed that the SNR efficiency is only ~1.4 times higher at 7T compared to 3T. This study also demonstrated high reproducibility and repeatability of sodium quantification using radial sequences with and without fluid suppression at 3T and 7T, which is comparable to other proton-based MR imaging techniques for assessing articular cartilage. This is a very promising result for further 3T studies on sodium imaging of cartilage.

## Cartilage Injury and Repair

### Background

Hyaline cartilage is avascular tissue. Thus, its response to injury differs from that of other tissues, and its intrinsic reparative capacity is very low [10, 45, 46]. In addition, the immobilized chondrocytes cannot migrate from healthy cartilage to the injury site. Mechanical injury can cause direct damage to the extracellular matrix, or the damage can be mediated by chondrocytes via reduction of biosynthetic activity and expression of matrix-degrading enzymes. Studies on animal models have shown that high impact loads to the joint can lead to a loss of tissue integrity, degradation of mechanical properties or cell death [47].

The result of the cartilage response to injury depends on several factors such as defect depth and location, the patient’s age, defect size, etc. Usual symptoms of cartilage injury are local pain, swelling, locking, pseudo-locking and catching. Isolated chondral defects were found in 4 % of arthroscopies, while a much higher percentage (40–70 %) of defects has been found in combination with ligament and/or meniscus injuries [46]. Thus, the cartilage health is very important for the health of the whole joint. Since untreated osteochondral defects in adults often lead to early onset of OA, symptomatic defects should be treated.

The goal of cartilage repair surgery techniques is to restore the cartilage surface and function, to allow pain-free motion of the joint and to prevent further cartilage degeneration by providing cartilage repair tissue that has the same composition, structure and mechanical properties as native articular cartilage [1]. Articular cartilage defects are currently treated with a number of different surgical interventions, which can be divided into three groups: (1) bone marrow-stimulating techniques, (2) auto-/allo-grafting techniques and (3) advanced cell-based repair techniques.

- Pridie drilling [48] and microfracture (MFX) [49] can be considered marrow-stimulating techniques. These procedures create multiple holes in the subchondral bone in the defect area to fill it with the material coming from the bone marrow. Ideally, the cells should differentiate into chondrogenic cells that produce cartilage [50]; however, these techniques usually result in the formation of fibrous repair tissue. Knutsen et al. [51] noted that most of the biopsies from repair tissue of patients 2 years after MFX were composed mainly of fibrocartilage tissue, which degenerates with time.
- Osteochondral grafting (mosaicplasty) [52] and periosteal grafting [53] rely on the filling of lesions with an autograft. In mosaicplasty, cylindrical osteochondral plugs are taken from non-load-bearing areas of an affected joint and placed into the osteochondral defect. Benefits of mosaicplasty are that the lesion is filled with mature hyaline cartilage and that it allows treatment of both osteochondral and chondral defects. Limitations of this technique are problematic production of a smooth convex cartilage surface, suboptimal reconstitution of the subchondral layer and usually insufficient lateral integration of the repair site [54].
- Advanced cell-based repair techniques such as autologous chondrocyte implantation (ACI) use donor-derived chondrocytes (mostly autografts) to reconstruct the cartilage defect. ACI was the first cell engineering approach to the treatment of cartilage lesions [55, 56]. In the third generation of ACI, such as in matrix-associated autologous chondrocyte transplantation (MACT), the pieces of cartilage are taken from a non-load-bearing donor site, and extracted chondrocytes are cultured in 3D scaffolds (matrices) in vitro for several weeks [57]. In the second step, the cultured cartilage is implanted into the chondral defect. Although the ACI can produce a hyaline-like repair tissue in some specimens, this tissue is not histochemically or morphologically identical to hyaline cartilage, and fibrocartilage can be found in some of the samples [58]. Although there were no significant differences in the histological quality of repair tissue between the patients after ACI and MFX 2 years after surgery, ACI patients more often showed hyaline-like repair tissue than MFX patients [51].

### Sodium MR Imaging of Cartilage Repair

Several studies used sodium MR imaging for the evaluation of tissue after cartilage repair surgery. The first sodium MR images of patients after MACT cartilage repair were published by Trattnig et al. [59•] in 2010. The authors measured 12 patients with a mean time of 56 months after MACT surgery in femoral cartilage and compared the results of sodium imaging at 7T with dGEMRIC (another GAG-sensitive MR technique) at 3T. The mean value of the magnetic resonance observation of cartilage repair tissue (MOCART) score [60], a scoring system for the evaluation of the morphological appearance of repair tissue, was 75 (range, 45–95). A 3D-GRE sequence optimized for sodium imaging was used together with a sodium-only birdcage knee coil. The sodium normalized values were significantly lower in repair tissue (mean ± standard deviation: 174 ± 53) than in normal-appearing reference cartilage (267 ± 42). Similarly, dGEMRIC measurements showed significantly lower postcontrast T1 values in repair tissue (510 ± 195 ms) than in reference cartilage (756 ± 188 ms). Moreover, a strong correlation was found between sodium imaging and dGEMRIC in MACT patients. The authors concluded that sodium imaging allows for differentiation of repaired tissue from native cartilage in patients after MACT repair without the application of contrast agent.

To validate and evaluate the potential of the gagCEST technique as a biomarker for GAG content in cartilage, Schmitt et al. [6] compared sodium imaging with gagCEST in five MFX and seven MACT patients. A strong correlation between sodium and gagCEST values proved the sensitivity of this method to GAG content in native cartilage and repair tissue.

**Fig. 3:**
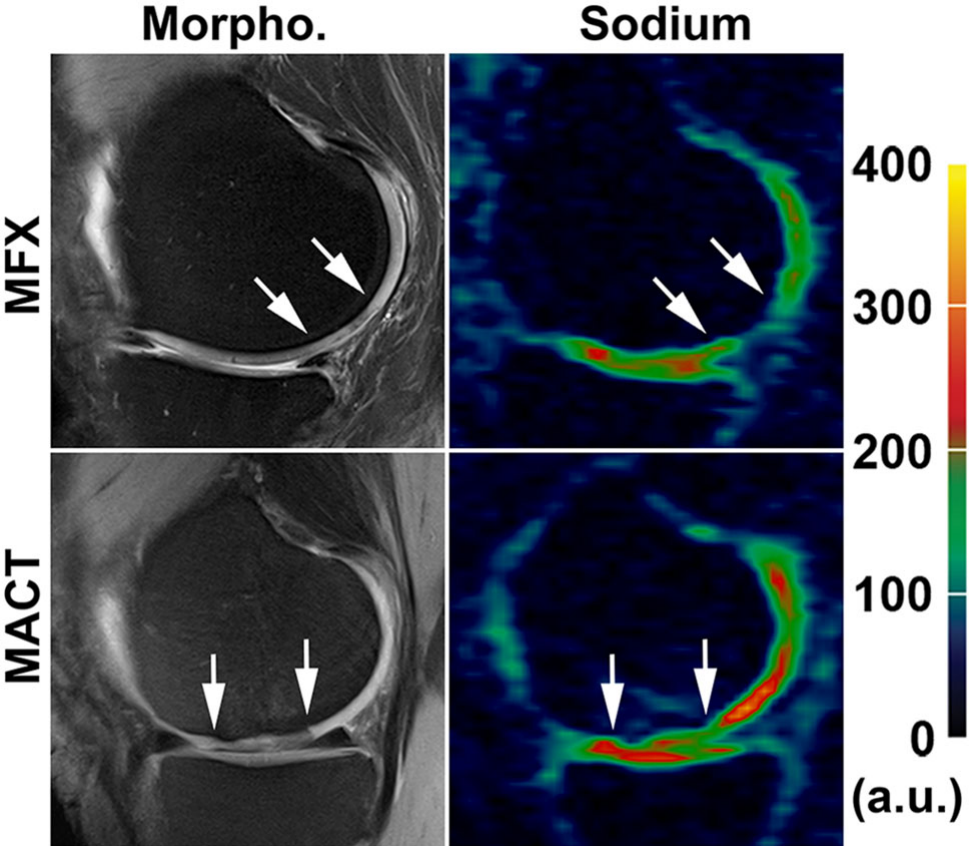
Proton density-weighted MR images with fat suppression (*left column*) and color-coded sodium 3D-GRE images (*right column*) from a patient after MFX treatment (*upper row*) and a subject after MACT surgery (*lower row*). The area of cartilage repair tissue is located between the *arrows*. *Color scale* represents the sodium signal intensity values

In the study by Zbyn et al. [61•], sodium MR imaging at 7T was used to evaluate repair tissue after two different types of cartilage repair techniques: bone marrow stimulation (BMS) techniques (Pridie drilling and MFX) and MACT. For more accurate comparison between repair techniques, each MACT patient was matched with one BMS patient according to age (mean, ~37 years), postoperative interval (mean, ~33 months) and defect location. Sodium images were measured with 3D-GRE sequences using a sodium-only birdcage knee coil, and ROIs were drawn in the reference cartilage and cartilage repair tissue. For both the BMS and MACT groups of patients, sodium normalized values were significantly lower in repair tissue than in reference cartilage. However, the main finding of this study was that sodium normalized values were significantly higher in MACT (210 ± 36) than in BMS (164 ± 31) repair tissue. On the other hand, the morphological appearance of the repair tissue, evaluated by the MOCART scoring system, was not different between BMS and MACT patients. The results suggest a higher GAG content and therefore repair tissue of higher quality in MACT than in BMS patients. Sodium imaging can distinguish between repair tissues with different GAG contents (Fig. 3) and thus can be useful for non-invasive evaluation of the performance of new cartilage repair techniques.

Due to the low resolution of sodium images, partial volume effects from surrounding tissues such as bone or synovial fluid (sodium concentration of 140–150 mmol/l) influence the accuracy of the sodium content measurements in cartilage. To minimize contamination from synovial fluid, triple quantum filtering techniques [16], inversion recovery (IR) methods [62] or relaxation-weighted sodium imaging [63] can be employed. The goal of the study by Chang et al. [64•] was to evaluate cartilage repair and native tissue using 7T sodium MR imaging with and without fluid suppression. After different cartilage repair procedures (e.g., MFX, osteochondral grafting, MACT, juvenile cartilage implantation) and with a median follow-up of 26 weeks (range 12–151 weeks), 11 patients were measured with a radial UTE sequence using a sodium-only birdcage knee coil. Fluid suppression was achieved using an IR preparation with an adiabatic inversion pulse [65]. The sodium concentration was calculated in repair tissue, adjacent native cartilage and native cartilage in the knee compartment not involved in surgery. Sodium concentrations were not significantly different between repair tissue and both types of native cartilage when using sequences without fluid suppression. This could be caused by heterogeneity of mean sodium concentrations in repair tissue due to the variety of repair procedures. However, when using fluid suppressed sequences, the mean sodium concentration in repair tissue (108.9 ± 29.8 mmol/l) was significantly lower compared to adjacent native cartilage (204.6 ± 34.7 mmol/l) or native cartilage in a different knee compartment (249.9 ± 44.6 mmol/l). Thus, fluid suppressed sodium imaging seems to be more accurate in the assessment of sodium concentration in repair tissue. Additionally, a significantly lower sodium concentration was found in adjacent native cartilage when compared to native cartilage in different knee compartments. This is in accord with in vitro studies that demonstrated that the number of viable chondrocytes increases with the distance from the site of injury [66].

## Osteoarthritis

### Background

Knee OA is associated with structural changes in the whole joint, including degradation of cartilage, ligaments, menisci, subchondral bone changes and synovial inflammation [67]. The diagnosis of OA is based on patient’s medical history, clinical symptoms (loss of function, pain) and radiographic evidence (joint space width) [67]. The goal of a treatment is to improve the knee function and relief from symptoms. The most frequent treatment of end-stage arthritis is knee joint replacement.

Although radiography is the current standard for evaluating structural modifications in joints in trials of potential disease-modifying OA drugs, it has many limitations (e.g., the sensitivity and precision) [68]. Morphological MR imaging is a non-invasive alternative that detects the presence of OA with high specificity compared to radiography or arthroscopy, and provides comprehensive evaluation of the whole joint [69]. MR imaging of knee OA includes assessment of the cartilage thickness and volume [70], synovitis, synovial fluid effusions, lesions in the bone marrow and meniscal damage. A loss of thickness in medial compartment cartilage is a sensitive quantitative parameter that correlates with radiographic joint space width and seems to be a strong predictor of the need for knee replacement [71]. Although MR imaging may be a viable alternative to radiography for the assessment of structural changes in knee OA and prediction of the knee replacement, both techniques are insensitive to biochemical changes in the cartilage, which occurs in early stages of OA before the morphological changes [3].

Detection of early events in OA, when the disease process is potentially reversible, is of major interest in cartilage imaging. GAG molecules have been considered to play a central role in cartilage homeostasis [72]. The equilibrium between synthesis and degradation of cartilage matrix molecules is altered in pathologic conditions [73]. For instance, increased levels of aggrecan 393ARGS fragments in the synovial fluid of patients with OA and knee injury have been proposed to reflect early pathology [74, 75]. Early stages of OA are suggested to be characterized by changes in the organization and composition of the extracellular matrix, such as a decrease in cartilage GAG content. However, the mechanisms associated with early OA are not entirely understood, and there are conflicting data on whether the GAG content is elevated [76–78], unchanged [79, 80] or decreased [81, 82] in early OA.

### Sodium MR Imaging of OA Cartilage

After validation in in vitro studies and in studies on animal models [83], sodium MR imaging was performed on volunteers and OA patients. Wheaton et al. [40] measured sodium images with UTE radial sequences in nine healthy asymptomatic volunteers and three patients with symptoms of early OA using a surface coil at 4T. The mean sodium content measured in the patellae of the nine healthy volunteers was 254 mmol/l, which corresponded to a mean FCD measurement of −182 mmol/l. Sodium maps for the subjects with symptoms of OA revealed cartilage regions with significantly lower FCD (−108 to −144 mmol/l) when compared to the FCD of healthy volunteers. These results suggest that sodium MR imaging might be a useful method for monitoring the changes in GAG content of OA cartilage.

The first 7T sodium MR images from OA patients were published by Wang et al. [84•]. Sodium images were acquired from five asymptomatic volunteers and five clinically diagnosed OA subjects using UTE radial sequences. The sodium concentration was calculated in three ROIs: patellar, medial femorotibial and lateral femorotibial cartilage. The mean sodium concentration in cartilage of volunteers ranged from 240 to 280 mmol/l. The sodium concentration in OA patients was significantly smaller, 30–60 % lower compared to volunteers. The authors concluded that sodium imaging may be a useful for physiologic OA imaging and clinical diagnosis. Unfortunately, due to very thin cartilage (especially in OA patients), low resolution and blurring in sodium images, the authors were not able to distinguish among patellar, femoral and tibial cartilage, and the evaluated ROIs probably included signal from both cartilage and synovial fluid.

**Fig. 4:**
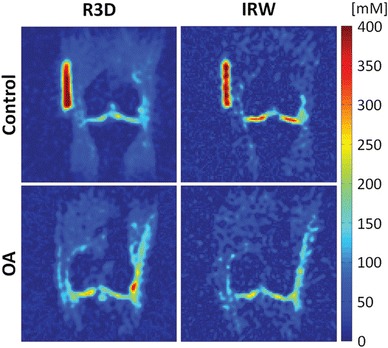
Sodium concentration maps from a control subject (*upper row*) and from a patient with OA (*lower row*). Sodium images were acquired with fluid suppression using IR (IRW) and without fluid suppression (R3D). Note that the difference in cartilage sodium concentration between the control and OA patient is higher with the fluid-suppressed radial sequence than with the radial sequence without fluid suppression. From [85••], with permission

To evaluate contamination from synovial fluid, Madelin et al. [85••] calculated the cartilage sodium concentration in 19 healthy volunteers and 28 OA patients using 7T sodium imaging with and without fluid suppression. The first eight volunteers and six patients were measured with a sodium-only birdcage knee coil; the rest of the subjects were measured with a homemade eight-channel double-tuned proton/sodium knee coil. Measurements with fluid suppression were achieved by using an IR preparation with an adiabatic inversion WURST pulse in the UTE radial sequence [65]. The cartilage sodium concentration was evaluated in the patellar, femorotibial lateral and femorotibial medial ROIs on four consecutive sodium maps. The mean sodium concentration over all cartilage ROIs measured with radial sequence without fluid suppression was similar between healthy subjects (192 mmol/l) and OA patients (174 mmol/l). When fluid suppression was applied, the difference between the mean sodium concentration over all cartilage ROIs in healthy subjects (~243 mmol/l) and in OA patients (~194 mmol/l) increased. The mean sodium concentration in cartilage from IR with WURST was found to be a significant predictor of OA and early OA (only patients with a Kellgren-Lawrence score of 1 or 2). An example of sodium MR imaging of OA cartilage with and without fluid suppression is shown in Fig. 4. Evaluation of the cartilage sodium concentration with fluid-suppressed MR imaging at 7T is a potential biomarker for OA.

## Conclusions

Recent studies proved that sodium MR imaging can directly, in a noninvasive and quantitative manner, assess the cartilage GAG content, which plays a central role in cartilage homeostasis in native and OA cartilage as well as in repair tissue. Since native reference cartilage (adjacent or more distant from the repair site) is available in sodium imaging of repair tissue, the sodium concentration is not the critical parameter, and ratios between native and repair sodium signal intensities can also be compared between patients. From this point of view, sodium imaging of OA cartilage is more demanding and requires quantification of the sodium concentration. Additionally, OA cartilage is thinner than native cartilage, and partial volume artifacts are therefore more pronounced in OA applications. This issue of sodium imaging can be overcome by introducing fluid-suppressed sequences, which allow evaluation of sodium signal from cartilage without contamination coming from synovial fluid [65]. Moreover, by using dual-tuned proton/sodium radiofrequency coils, sodium imaging can be combined with other MR techniques (e.g., morphological imaging, T2 mapping, diffusion-weighted imaging) to assess other cartilage components (e.g., water content, collagen matrix). Such a combination of biomarkers might provide more accurate insight into cartilage degeneration or maturation of repair tissue. However, acquisition times of sodium imaging must be reduced to acquire a complete imaging protocol in less than 1 h.

Previous in vitro studies showed changes in T1 and T2* relaxation times of cartilage after enzymatically induced (trypsin, papain) PG depletion [34–36]. Thus, different T1 and T2* relaxation times can also be expected in OA cartilage and repair tissue. The general limitation of the sodium concentration quantification is the use of sodium T1 and T2* values from native cartilage also for quantification of the concentration in OA cartilage and in repair tissue, which might result in bias from ‘true’ sodium concentrations. To overcome this limitation might be difficult as the measurement of sodium relaxation times is more time consuming than morphological sodium imaging. However, the information on cartilage relaxation times might improve the sensitivity and specificity of sodium imaging in evaluating degenerative changes in cartilage.

Sodium MR imaging is a challenging method. However, new technical developments in the recent decade have enabled transferring this technique from in vitro to pre-clinical in vivo studies. The advent of whole-body ultra-high-field MR systems [43, 86], dedicated radiofrequency phase-array coils [87] and optimized MR sequences [65] has provided higher SNR, higher spatial resolution in the images and/or shorter measurement times. Moreover, compressed sensing applications for the acceleration of sodium imaging acquisition with undersampling are very promising [88, 89]. However, more research is necessary on both software and hardware to translate sodium MR imaging of cartilage into clinical practice, preferably to 3T MR systems.
